# Bridging Fear of Negative Evaluation and Cognitive Emotion Regulation Strategies: A Network Perspective on the Roles of Family Functioning and Self‐Control

**DOI:** 10.1155/da/1391628

**Published:** 2026-05-29

**Authors:** Xingyu Wang, Yaxin Li, Dongdong Zhang, Hongjuan Chang

**Affiliations:** ^1^ Department of Nursing, Medical College, Wuhan University of Science and Technology, Wuhan, Hubei, China, wust.edu.cn

## Abstract

**Background:**

In the context of intensifying social pressure, college students’ high sensitivity to negative evaluations significantly affects their mental health. This study uses network analysis to explore the network structure characteristics of Chinese college students’ fear of negative evaluations, family functioning, and self‐control, as well as their conditional associations with cognitive emotion regulation strategies (CERSs).

**Method:**

A convenience sampling method was used to conduct a cross‐sectional online questionnaire survey of 5527 college students enrolled at three universities in Wuhan. The study employed the Fear of Negative Evaluation Scale, Family Care Scale, Self‐Control Scale, and CERSs Scale. Data analysis was conducted using SPSS 26.0 and R Studio 4.5.1 software to construct a psychological trait network, identify core and bridge nodes, and analyze their associations with CERSs (adaptive and maladaptive).

**Results:**

BFNE9 (continuous concern about others’ impressions) had the highest expected influence (EI); FC4 (family emotional support) and FC2 (family partnership) had high centrality, highlighting the core role of family functioning. Bridge analysis indicated that BS4 (difficulty controlling attention) was the strongest bridge node connecting different psychological dimensions, followed by BS1 (self‐control) and BS7 (impulsivity). Flow network analysis further revealed that BFNE12 (fear of saying the wrong thing) exhibited the strongest positive association for both adaptive (ER1) and nonadaptive (ER2) CERSs, suggesting that expressive evaluation anxiety is strongly connected with multiple components within the emotional regulation network.

**Conclusion:**

This study used psychological network analysis to reveal the complex interactive structure between fear of negative evaluation, family functioning, and self‐control and highlighted the central role of expressive evaluation anxiety (BFNE12) within the network. The results provide a new perspective and theoretical basis for understanding the psychological mechanisms related to social anxiety in college students and for informing future research on potential intervention directions. However, given the cross‐sectional design, causal interpretations cannot be inferred.

## 1. Introduction

With the growing intensity of social competition, mental health problems among university students have been rising steadily [1]. As individuals in early adulthood, this group is situated at a stage where psychological development and socialization demands are especially pronounced. Simultaneously, they must cope with multiple pressures stemming from academic performance, interpersonal relationships, and future career planning [2]. These overlapping challenges place university students at a heightened risk of psychological maladjustment. According to the World Health Organization (WHO), nearly 31% of adolescents worldwide experience some degree of psychological distress, with anxiety and depression being the most prevalent conditions [3, 4]. Understanding and addressing the psychological challenges faced by this population have thus become a pressing issue for contemporary psychological research and practice.

Fear of negative evaluation refers to a persistent concern or apprehension about receiving unfavorable judgments from others during social interactions [5]. This concern is not limited to specific social situations. Nonetheless, it can extend broadly to academic, occupational, and other life domains and is associated with both mental health and social functioning [6]. From a social–cognitive perspective, adolescence through early adulthood represents a developmental period in which cognitive capacities such as perspective‐taking undergo rapid growth. This developmental shift increases sensitivity to external evaluations, thereby heightening vulnerability to anxiety and stress across diverse social contexts [7]. Sustained levels of fear of negative evaluation are associated with maladaptive outcomes, including social avoidance, heightened feelings of isolation, and the emergence of depressive symptoms. Previous studies have consistently identified fear of negative evaluation as a central psychological factor and have been linked to the onset and maintenance of social anxiety disorder [8]. When facing evaluative stress, the strategies individuals use to regulate their emotions play a crucial role in their psychological adjustment. Cognitive emotion regulation strategies (CERSs) refer to the cognitive processes individuals use to manage their emotional responses following adverse or stressful experiences [9]. These strategies are often classified into adaptive forms (e.g., positive reappraisal and seeking social support) and maladaptive forms (e.g., rumination and self‐blame) [10]. A recent study, using a sample of university students, employed network analysis to investigate the structural role of different CERSs within the broader psychological system. The findings indicated that catastrophizing and self‐blame emerged as central nodes in the symptom network, showing strong associations with both anxiety and depression. These results underscore the detrimental impact of maladaptive regulation strategies on mental health [11].

However, existing research has primarily focused on the internal cognitive mechanisms of emotion regulation, with relatively little attention paid to external factors that may shape these processes. Prior evidence suggests that social support plays a vital role in influencing cognitive emotion regulation [12, 13]. Family functioning, as a key support system during early socialization, refers to the extent to which family members provide emotional understanding, acceptance, and encouragement [14]. Higher levels of family support facilitate the development of healthy regulation patterns and strengthen resilience against evaluative threats. Meanwhile, low family functioning can diminish regulatory resources and increase vulnerability to social evaluative stress [15, 16]. Thus, Ye et al. [17], using network analysis among Chinese vocational college students, identified “partnership” and “emotional response” as the most central nodes linking anxiety and depression, underscoring the regulatory role of family support structures in maintaining mental health. Additional studies further demonstrate that adolescents who perceive low family functioning are more likely to exhibit social anxiety and school refusal behaviors. Meanwhile, higher levels of family support may reduce difficulties in emotion recognition and indirectly alleviate social anxiety among university students [18, 19]. Collectively, this body of evidence highlights the protective role of family functioning in maintaining a psychological well‐being. In the Chinese cultural context, family functioning is also closely tied to the concept of “mianzi” (face), where maintaining social harmony and avoiding negative evaluation are highly valued, potentially amplifying the role of family support in regulating evaluative concerns.

Simultaneously, individual psychological resources such as self‐control play an equally critical role in both cognitive emotion regulation and broader psychological adjustment. Self‐control generally refers to the ability to regulate emotions and behaviors in the face of temptation, impulsivity, or stress, thereby achieving long‐term goals [20]. High levels of self‐control are associated with more adaptive emotion regulation strategies and reduced risk of anxiety and depression. In contrast, low self‐control is often associated with emotional dysregulation, impulsive reactions, and increased vulnerability to psychological problems [21–23]. Recent findings further suggest that self‐control not only buffers the impact of negative emotions but may also be related to the choice of regulation strategies, thereby moderating the pathway between Brief Fear of Negative Evaluation Scale (BFNE) and mental health outcomes [24–27]. In research examining problematic smartphone use and exercise procrastination, self‐control was identified as the critical bridging variable linking the two, suggesting that it may constitute a common underlying mechanism for a wide range of maladaptive behaviors and emotional difficulties [28]. Additionally, studies exploring the relationship between internet addiction and CERSs have shown, through network analysis, that catastrophizing and other‐blame serve as bridging nodes between alexithymia and regulation strategies [29]. These findings underscore the sensitivity and explanatory value of network approaches in detecting cross‐system interactions.

Drawing on emotion regulation theory [30, 31] and social support theory [32, 33], the present study examined the interplay among BFNE, family functioning (FC), self‐control (BS), and CERSs (CERQ) through item‐level and flow network analyses. Based on prior empirical findings, some hypotheses were specified as confirmatory, whereas others were considered exploratory given the limited existing evidence at the item level. In the item‐level network, we hypothesized that Ha1a (confirmatory): items reflecting evaluative sensitivity in BFNE—BFNE1: worry about being evaluated and BFNE9: concern about others’ impressions—would show strong associations with key indicators of self‐control and family support (BS1: self‐control and FC4: emotional closeness). Ha1b (confirmatory): supportive items within FC—FC2: partnership and FC4: emotional closeness—would emerge as central nodes that enhance self‐control through their interconnections. In the flow network, we expected that Hb1 (exploratory): anxiety‐ and avoidance‐related items of BFNE—BFNE1 and BFNE12. Specifically, the fear of saying something wrong would be directly linked to maladaptive CERS (rumination and self‐blame). Hb2 (confirmatory): supportive items within FC (FC4, FC5: problem solving) would predict adaptive CERS (positive reappraisal and seeking support). Hb3 (exploratory): indicators of self‐discipline and goal orientation in BS (BS3 and BS5) would function as protective factors, reducing reliance on maladaptive strategies. Compared to traditional approaches, such as regression or structural equation modeling, the use of item‐level and flow networks enables a finer‐grained mapping of within‐ and between‐construct associations. They also help identify key bridge items that directly connect to adaptive and maladaptive regulation strategies. Altogether, these methods provide a novel framework for understanding the mechanisms linking BFNE, family functioning, and self‐control to emotion regulation in university students.

## 2. Methods

### 2.1. Procedure and Participants

Data collection was conducted in March 2024 through convenience sampling of college students from three universities in Wuhan, Hubei Province. Participants completed an online questionnaire distributed via a secure platform. A total of 5750 questionnaires were distributed. Responses with completion times of ≤5 min or ≥60 min, as well as submissions showing clear duplication, were excluded from the analysis. The lower threshold (≤5 min) was determined based on preliminary testing and data quality screening conducted prior to the formal survey, which indicated that excessively short completion times were likely to reflect insufficient engagement or inattentive responding, thereby compromising data reliability. After applying these criteria, 5527 valid questionnaires were retained, yielding an effective response rate of 96.12%. The final sample comprised 1610 male students (29.13%) and 3917 female students (70.87%), which reflects the typical gender distribution in medical universities in China, where female students are overrepresented. Nevertheless, given the gender imbalance, the findings should be interpreted with caution regarding potential sex differences. Ethical approval for this study was obtained from the Ethics Committee of Wuhan University of Science and Technology (Approval Number: 2023177). Given the use of convenience sampling from three universities, potential selection bias cannot be ruled out, and the generalizability of the findings may be limited. All participants were informed about the study objectives and provided written informed consent prior to participation. Reporting of this study followed the STROBE Statement checklist (Supporting Information 1: S1).

### 2.2. Measures

Participants provided information on gender, age, educational background, place of origin, whether they were only children, academic ranking, frequency of exercise, family type, and parenting style. Gender was not included as a covariate nor were network invariance analyses conducted; therefore, potential gender differences in network structure remain to be examined in future research.

Fear of negative evaluation was assessed using the Chinese revised version of the BFNE, adapted by Chen et al. [8]. The scale consists of 12 items, including both positively and negatively worded statements reflecting concerns about others’ evaluations. Negatively worded items were reverse‐coded prior to the analysis. The scale is scored using a 5‐point Likert scale (1 = completely disagree and 5 = completely agree), with a total score ranging from 12 to 60 points. Higher scores indicate that individuals are more concerned about whether they have left a good impression on others and are more sensitive to negative evaluations from others. Previous studies in Chinese university samples have reported acceptable psychometric properties of this version, including satisfactory internal consistency, construct validity, and a stable factor structure. The Cronbach’s α coefficient for the questionnaire in this study was 0.737, and the split‐half reliability was 0.752.

The Brief Self‐Control Scale (BSCS) is used to assess an individual’s ability to inhibit impulsive behavior and their level of behavioral self‐discipline in daily life. The Chinese version of the Self‐Control Scale includes two dimensions: impulse control and self‐discipline, with a total of seven items [34]. The scale uses a 5‐point Likert scale, ranging from 1 (completely disagree) to 5 (completely agree), with items 2, 4, 6, and 7 being reverse‐scored. A higher total score indicates a stronger self‐control ability. In this study, the Cronbach’s α coefficient for this scale was 0.83.

The Family APGAR Questionnaire (APGAR) is used to assess an individual’s subjective satisfaction with their family functioning [35]. The questionnaire consists of five items, all of which are scored positively, reflecting family functioning in five dimensions: adaptation, cooperation, growth, emotion, and intimacy. A three‐point scoring method is used, ranging from 0 (almost never) to 2 (often), with a total score of 0–10 points. A score of 7–10 indicates good family functioning, 4–6 indicates moderate impairment, and 0–3 indicates severe impairment. A higher total score indicates better family functioning. In this study, Cronbach’s α coefficient for this scale was 0.90.

The Chinese Version of the Cognitive Emotion Regulation Questionnaire (CERQ‐C) was developed by Garnefski and introduced to China by Dong Guangheng et al. in 2008, with the aim of assessing the CERSs individuals employ when faced with negative events. The questionnaire consists of 9 subscales, each comprising 2 items, totaling 18 items, categorized into adaptive and nonadaptive cognitive regulation strategies. It employs a 5‐point Likert scale, ranging from 1 (never) to 5 (always), with higher scores indicating greater frequency of strategy use [36]. In this study, Cronbach’s alpha coefficient for the scale was 0.912, with alpha coefficients for each subscale ranging from 0.79 to 0.89, indicating that the tool has high internal consistency and good reliability. All instruments employed Likert‐type or ordinal response formats. Detailed item descriptions and corresponding node abbreviations used in the network analyses are presented in Supporting Information 2: S2.

### 2.3. Data Analysis

#### 2.3.1. Network Estimation

Data analyses were conducted using R version 4.5.1 and RStudio. Network estimation and visualization were performed using the qgraph package (version 1.9.2). A Gaussian graphical model (GGM) was used to estimate the conditional association structure among nodes. Prior to network estimation, all variables were transformed using the huge.npn() function from the huge package, with npn.func = “shrinkage,” to reduce skewness and improve the approximation to multivariate normality. Based on the transformed data, a Spearman correlation matrix was computed using pairwise complete observations and used as the input for network estimation. The network was estimated using the EBICglasso procedure, with the extended Bayesian information criterion (EBIC) hyperparameter γ (tuning parameter) set to 0.5 [37, 38]. Under this framework, the regularization parameter was selected automatically by minimizing the EBIC, thereby balancing model sparsity and goodness of fit. Given that most variables were measured using Likert‐type or ordinal response formats, treating them as approximately continuous within the GGM framework may still introduce estimation bias; therefore, the results should be interpreted with caution.

The network estimation proceeded in two stages. In the first stage, an item‐level network was constructed based on all items from the scales measuring fear of negative evaluation, family functioning (FC), and self‐control (BS). This network was used to examine the structural relationships among the three constructs and to identify the central and bridge nodes. Based on this structure, node predictability was computed using the mgm package (version 1.2–12). Predictability quantifies the proportion of variance in each node explained by its direct neighbors and was displayed as a ring around each node, highlighting which items were more strongly determined by their neighboring nodes within the network structure [39].

In the second stage, we extended the analysis to incorporate CERSs. Specifically, the two higher‐order dimensions of CERS—adaptive and maladaptive strategies—were introduced as dimension‐level nodes into the item‐level network to construct a flow network. In this framework, the two CERS dimensions were treated as reference nodes, allowing us to visualize which BFNE, FC, and BS items were directly conditionally associated with adaptive and maladaptive regulation dimensions within the estimated network. This approach allowed us to map how fear of negative evaluation, family functioning, and self‐control were conditionally associated with different regulation strategy dimensions. Flow network visualization was implemented using the flow function in the qgraph package [40], which provided an interpretable display of the direct conditional associations surrounding the selected reference nodes. Importantly, this visualization does not imply temporal, causal, or predictive ordering.

#### 2.3.2. Centrality and Stability

To identify influential nodes and their potential roles within the network, we conducted both centrality and bridge analyses. The primary index was expected influence (EI), which reflects the overall strength of a node’s connections by summing the edge weights of its directly connected edges while preserving the sign of each edge. This provides an estimate of a node’s net influence within the network. EI was computed using the networktools package and applied to both the item‐level network and the flow network [41–43]. To examine bridging effects across different psychological domains—fear of negative evaluation, family functioning, and self‐control—we computed bridge EI (BEI) within the item‐level network. BEI quantifies the extent to which a node links constructs from different communities, thereby identifying potential cross‐domain associations [44]. The stability of centrality indices and edge weights was evaluated through nonparametric bootstrap resampling with 1000 iterations using the bootnet package. First, 95% confidence intervals for edge weights were generated to assess estimation accuracy. Second, the case‐dropping bootstrap method was applied to calculate the correlation stability (CS) coefficient for EI and BEI. A CS coefficient greater than 0.50 was considered evidence of high stability and reliable interpretability of the centrality indices [45, 46].

Given that the items from the Family APGAR, BSCS, and BFNE were measured using Likert‐type or ordinal response formats, an ordinal‐data sensitivity analysis was conducted to examine the robustness of the item‐level network results across estimation methods. Specifically, under the same EBICglasso framework with the EBIC hyperparameter γ set to 0.5, an ordinal‐based correlation matrix was estimated using the cor_auto function, and an alternative ordinal‐based network was subsequently constructed. The ordinal‐based network was then compared with the primary item‐level network in terms of overall edge‐weight structure, as well as the rankings of EI and BEI, in order to evaluate the stability and robustness of the main findings.

## 3. Results

### 3.1. Study Sample

This study included 5527 college students (29.13% male and 70.87% female). In terms of educational level, 1065 were junior college students, 3786 were undergraduates, and 676 were postgraduate students. Additionally, 12.97% of participants were only children, and 38.86% reported left‐behind experiences. Detailed demographic and family‐related characteristics are presented in Table 1. For the main study variables, students scored an average of 40.08 (SD = 1.93) on the Fear of Negative Evaluation scale, which is higher than the theoretical median of 36. A total of 3153 students (BFNE ≥ 36) scored above the theoretical median, indicating relatively higher levels of BFNE within the sample. The mean score for family care was 2.32 (SD = 0.63) and for self‐control was 3.05 (SD = 0.90). Item‐level scores and their predictability indices are reported in Table 2.

**Table 1:** Demographic characteristics of the college student sample (*n* = 5527).

Variable	Category	*N* (%)
Gender		
	Male	1610 (29.13)
	Female	3917 (70.87)
Education level		
	Junior college	1065 (19.27)
	Undergraduate	3786 (68.50)
	Postgraduate and above	676 (12.23)
Place of residence		
	Urban	2900 (52.47)
	Rural	2627 (47.53)
Only child		
	Yes	717 (12.97)
	No	4810 (87.03)
Academic ranking		
	Top 10	793 (14.35)
	Upper‐middle	1264 (22.87)
	Middle	2643 (47.82)
	Lower‐middle	619 (11.20)
	Bottom 10	208 (3.76)
Exercise frequency (min/week)		
	60 min–90 min	1974 (35.72)
	91 min–120 min	2454 (44.40)
	≥121 min	1099 (19.88)
Sleep quality		
	Good	3170 (57.35)
	Average	2284 (41.32)
	Poor	73 (1.33)
Family type		
	Nuclear family	2000 (36.19)
	Extended family	320 (5.79)
	Single‐parent family	50 (0.90)
	Joint family	91 (1.65)
	Reorganized family	100 (1.81)
	Intergenerational family	2966 (53.66)
Left‐behind experience		
	Yes	2148 (38.86)
	No	3379 (61.14)
Parenting style		
	Strict	1331 (24.08)
	Spoiling	92 (1.66)
	Neglectful or indifferent	218 (3.94)
	Abusive	52 (0.94)
	Inconsistent	356 (6.44)
	Warm	3478 (62.92)
Monthly household income per capita		
	>6000	696 (12.59)
	4000–5999	1160 (20.99)
	2000–3999	1917 (34.68)
	<2000	1754 (31.74)

**Table 2:** Descriptive statistics of FC5, BS7, and BFNE12 items.

Item abbreviation	Items context	Mean	SD	Predictability
FC1	Adaptation	1.189	0.620	0.570
FC2	Partnership	1.208	0.672	0.660
FC3	Growth	1.382	0.613	0.606
FC4	Affection	1.339	0.645	0.712
FC5	Resolve	1.465	0.616	0.639
BS1	Self‐control	3.556	0.877	0.442
BS2	Impulsivity	2.679	1.133	0.348
BS3	Discipline	2.974	0.949	0.445
BS4	Distractibility	3.156	0.973	0.311
BS5	Goal orientation	3.428	0.881	0.467
BS6	Temptation‐prone	2.781	1.033	0.450
BS7	Impulsiveness	2.806	0.990	0.393
BFNE1	Worry about judgment	3.644	0.525	0.546
BFNE2	Indifference to negative views	3.149	0.640	0.435
BFNE3	Fear of criticism	3.394	0.596	0.607
BFNE4	Unconcerned with impressions	2.914	0.570	0.470
BFNE5	Fear of disapproval	3.355	0.481	0.662
BFNE6	Fear of mistakes	3.172	0.481	0.659
BFNE7	Unbothered by opinions	3.294	0.462	0.372
BFNE8	Worry in conversations	3.479	0.623	0.673
BFNE9	Constant concern about impressions	3.505	0.570	0.704
BFNE10	Unfazed by evaluation	2.990	0.592	0.399
BFNE11	Overly concerned with opinions	3.496	0.454	0.666
BFNE12	Fear of saying something wrong	3.688	0.644	0.641

### 3.2. Network Structure

**Figure 1:**
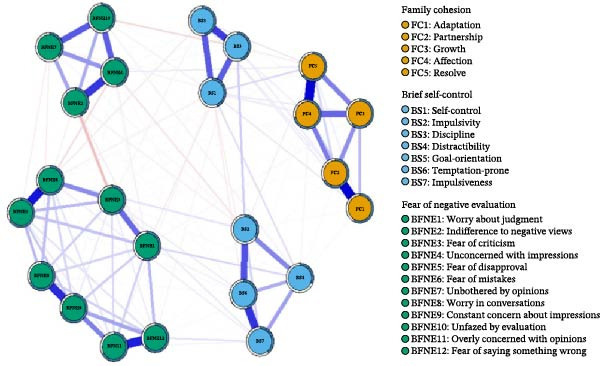
Network structure of negative evaluation fear, family functioning, and self‐control among college students.

The estimated network structure of Chinese university students’ fear of negative evaluation, family functioning (FC), and self‐control (BS) is presented in Figure 1. In the 24‐node network, 148 of the 276 possible edges were nonzero, indicating a moderately dense network structure (density = 0.536) [47]. The network exhibited clear modularity, with items from each construct forming relatively distinct clusters. The strongest within‐domain connections among family functioning items were FC1 (adaptation)–FC2 (partnership; weight = 0.451) and FC4 (affection)–FC5 (resolve; weight = 0.447). Within the BFNE community, the most prominent edges were BFNE5 (fear of disapproval)–BFNE6 (fear of mistakes; weight = 0.413), BFNE11 (overly concerned with opinions)–BFNE12 (fear of saying something wrong; weight = 0.398), and BFNE8 (worry in conversations)–BFNE9 (constant concern about impressions; weight = 0.375). Within the self‐control community, the strongest connections were BS6 (temptation‐prone)–BS7 (impulsiveness; weight = 0.363) and BS3 (discipline)–BS5 (goal orientation; weight = 0.343).

In contrast, cross‐domain associations were comparatively weak. The strongest cross‐domain edge was observed between BS4 (distractibility) and BFNE1 (worry about judgment; weight = 0.070), followed by FC5 (resolve)–BS1 (self‐control; weight = 0.061) and BS3 (discipline)–BFNE10 (unfazed by evaluation; weight = −0.061). Overall, these findings suggest that fear of negative evaluation, family functioning, and self‐control formed internally coherent subnetworks, whereas the connections across domains were relatively sparse and modest in magnitude.

**Figure 2:**
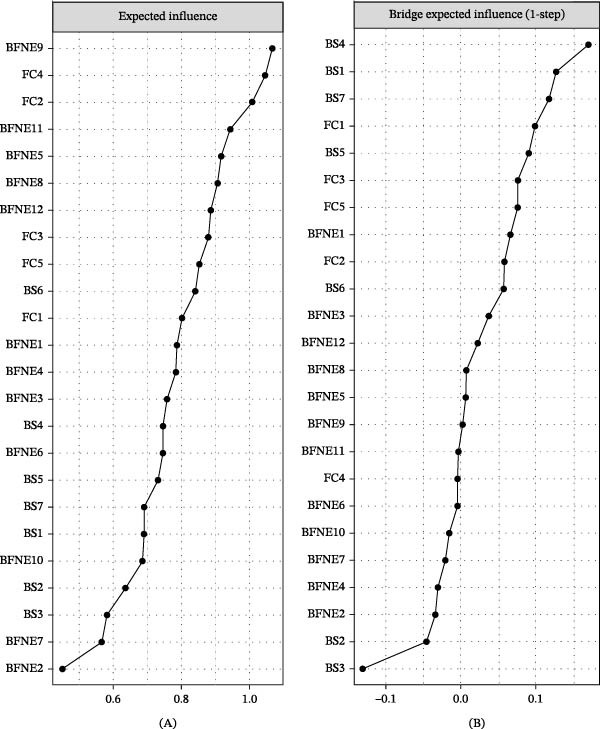
Expected influence of BFNE112, BS17, and FC15 and bridge expected influence. *Note:* Entries are listed in descending order from top to bottom, from the node with the highest centrality value to the node with the lowest centrality value. (A) Expected influence (EI) of nodes from the BFNE, BS, and FC scales, sorted in descending order (highest to lowest, top to bottom). (B) One‐step bridge expected influence (BEI) of nodes, sorted in descending order (highest to lowest, top to bottom).

The EI and BEI of each node in the network of college students’ fear of negative evaluation, family care, and self‐control are shown in Figure 2. Across the entire network, BFNE9 (constant concern about impressions) exhibited the highest EI. FC4 (affection) and FC2 (partnership) also demonstrated significantly higher EI values than other nodes (Figure 2A), indicating that these nodes were strongly connected within the network structure. In terms of BEI, BS4 (distractibility) was the most prominent, followed by BS1 (self‐control) and BS7 (impulsiveness). These three nodes served as key bridges connecting different psychological dimensions (Figure 2B).

### 3.3. Network Stability

**Figure 3:**
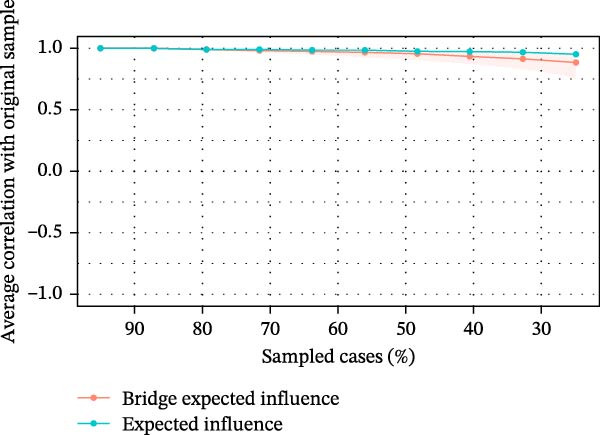
Stability of centrality and bridge centrality metrics using case‐drop bootstrap.

The results of the case‐dropping bootstrap analysis are shown in Figure 3. The CS coefficients for EI and BEI were both 0.75, indicating good stability and supporting the interpretability of these centrality indices. Supporting Information 3: S3 presents the bootstrap 95% confidence intervals for edge weights. Overall, confidence intervals tended to be narrower for stronger edges and wider for weaker edges, suggesting that weaker connections were estimated with greater uncertainty. Supporting Information 3: S3 presents the bootstrap difference test for edge weights, indicating that some edge weights differed significantly from others, whereas many pairwise differences were not statistically significant. Supporting Information 3: S3 displays the bootstrap difference test for node EI values, showing that some nodes had significantly higher EI values than others, although many node pairs did not differ significantly.

### 3.4. Ordinal‐Data Sensitivity Analysis

To further evaluate the robustness of the network results when treating items as Likert‐type or ordinal data, an additional sensitivity analysis based on cor_auto was conducted. Under the same EBICglasso framework, the item‐level network was re‐estimated using the ordinal‐based correlation matrix. The results indicated a high degree of consistency between the ordinal model and the primary analysis in terms of the overall edge‐weight structure, with a Spearman rank correlation of 0.894 and a Pearson correlation of 0.989 across all edge weights. Regarding node centrality, the EI rankings were highly consistent between the two models, with a Spearman rank correlation of 0.954 and a Pearson correlation of 0.964. Similarly, for bridge centrality, the BEI rankings also showed strong agreement, with a Spearman rank correlation of 0.891 and a Pearson correlation of 0.918.

In the primary model, BFNE9 (persistent concern about others’ impressions) exhibited the highest EI value. This finding was replicated in the ordinal model, where BFNE9 remained the most central node, indicating the robustness of its central role. In addition, FC4 (affection) and FC2 (partnership) consistently showed relatively high EI values across both models, suggesting that family functioning items maintained a central position within the overall network. Notably, in the ordinal model, the centrality of FC5 (problem solving) further increased, indicating that the problem‐solving dimension of family functioning may play a particularly important role.

With respect to bridge centrality, both the primary and ordinal models identified BS4 (distractibility) as the node with the highest BEI, supporting its role as a key bridge linking fear of negative evaluation, family functioning, and self‐control. Meanwhile, nodes such as BS1, BS7, FC3, and FC5 also exhibited relatively high bridge centrality in the ordinal model, although their exact rankings showed some variation compared to the primary model. This suggests that, aside from BS4, the relative positions of secondary bridge nodes may be somewhat sensitive to the estimation method.

In terms of the strongest edges, both models showed that the most prominent connections were primarily located within the same constructs. These were particularly concentrated among family functioning items (e.g., FC2–FC1 and FC5–FC4), fear of negative evaluation items (e.g., BFNE6–BFNE5, BFNE12–BFNE11, and BFNE9–BFNE8), and self‐control items (e.g., BS7–BS6 and BS5–BS3), indicating good overall structural stability of the network.

**Figure 4:**
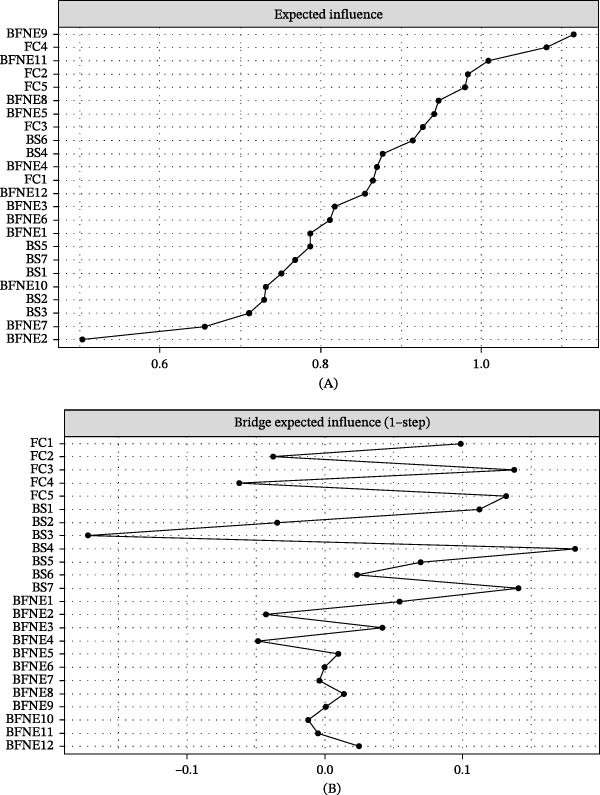
Expected influence of entries BFNE112, BS17, and FC15 (A) and bridge expected influence (1‐step) (B) in the ordinal‐data sensitivity analysis.

Taken together, the main findings of the item‐level network, particularly the central role of BFNE9 and the bridging function of BS4, remained stable under the ordinal data specification. This suggests that these results are not merely artifacts of treating Likert‐type items as approximately continuous variables (details are shown in Figure 4).

### 3.5. Flow Network

**Figure 5:**
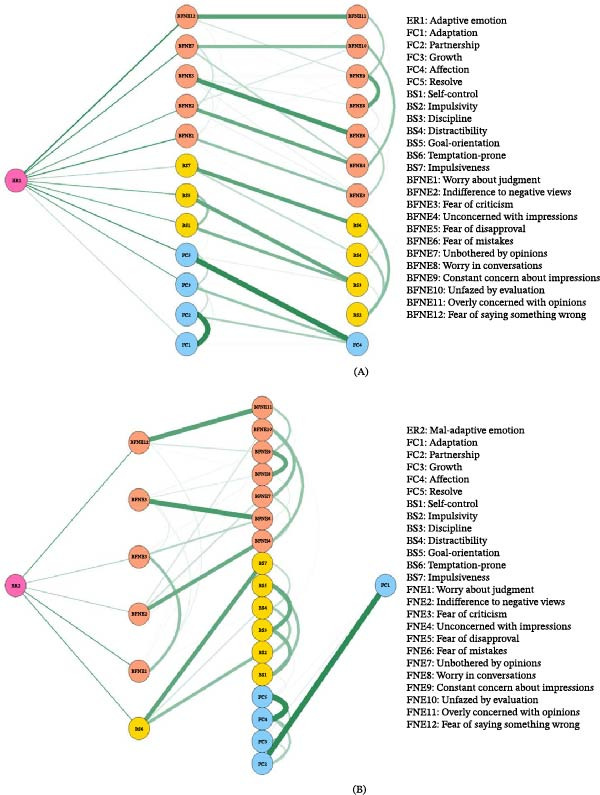
(A) Adaptive cognitive emotional regulation strategy flow network. (B) Flow network of maladaptive cognitive emotional regulation strategies.

Figure 5 presents the flow network structure of adaptive emotion regulation strategies (ER1) in relation to fear of negative evaluation, self‐control (BS), and family functioning (FC) (Figure 5A). In this structure, 11 nodes showed direct conditional associations with ER1, including five BFNE variables (BFNE1, BFNE2, BFNE5, BFNE7, and BFNE12), two self‐control variables (BS1 and BS5), and four family functioning variables (FC1, FC2, FC3, and FC5). The remaining nodes were indirectly connected through intermediate nodes. The edge between ER1 and BFNE12 (fear of saying something wrong) had the largest average weight (0.137), highlighting its strong association with adaptive emotion regulation strategies. In the flow network based on maladaptive emotion regulation strategies (ER2) (Figure 5B), ER2 was directly connected to six nodes: BFNE1, BFNE2, BFNE3, BFNE5, BFNE12, and BS6. Among these, the edge between ER2 and BFNE12 was also the strongest, with an average weight of 0.094, suggesting that fear of saying something wrong (BFNE12) was strongly associated with both adaptive and maladaptive emotion regulation strategies.

## 4. Discussion

To our knowledge, few studies have employed an item‐level network analytic approach to systematically examine the complex interplay among three core psychological constructs among Chinese university students. These include fear of negative evaluation, family functioning, and self‐control. By further incorporating a flow network model, the study described the conditional association structure between these constructs and CERSs. From a whole‐network perspective, the analysis not only identified critical nodes and bridging elements but also highlighted their relative positions within the broader emotion regulation system. In doing so, this work extended the current understanding of association patterns related to psychological adaptation in university students, offering a more nuanced view of how individual vulnerabilities and protective resources are interconnected. Given the cross‐sectional design and the use of network analysis, the findings should be interpreted as reflecting conditional association patterns rather than causal mechanisms.

The results showed that BFNE9 (persistent concern about others’ impressions) was the most central node in the network, indicating that it was strongly connected to fear of negative evaluation, family functioning, and self‐control among college students. A constant concern about others’ opinions reflects a heightened sensitivity to evaluation, which has been associated with social anxiety and related difficulties in behavioral regulation, interpersonal functioning, and emotional coping [48–50]. This finding is consistent with earlier network analyses of adolescent social anxiety. For example, a study of Chinese adolescents found that concerns about others’ evaluations occupied central positions in the network and were closely related to problematic smartphone use, avoidance behaviors, and other psychological difficulties [51]. This supports the central role of BFNE9 identified in the present study. From a correlational perspective, one recent study found that concerns about negative evaluation were associated with social avoidance, which indirectly contributed to psychological distress [16]. This suggests that BFNE9 represents a cognitive hallmark of social anxiety that is associated with multiple domains, including family dynamics and self‐regulation, and other dimensions of psychological functioning. Similarly, individuals with a stronger fear of negative evaluation are more likely to interpret ambiguous social cues as negative feedback, which further increases their vigilance in social situations [52]. Such cognitive biases may help explain why BFNE9 is strongly connected across domains in emotion regulation, cognitive processing, and behavioral expression [53]. Furthermore, FC4 (affection) and FC2 (partnership) were ranked second and third in EI within the network. This finding is consistent with previous research, which has demonstrated that emotional support and cooperative family dynamics are essential for psychological adjustment. A study conducted in Spain, exploring the relationship between family functioning and social anxiety, found that adolescents from families with higher levels of emotional support demonstrated stronger emotion‐regulation capacities and reduced negative reactivity under stress, highlighting the buffering function of family affection [14]. Similarly, a study of undergraduates from three Chinese universities showed that positive family communication and support enhanced self‐esteem and reduced sensitivity to negative evaluation in emerging adults. This thereby alleviates social anxiety [15]. Both studies are consistent with the present results, emphasizing the protective role of family functioning. Additionally, a study of Chinese vocational college students using network analysis found that family partnership was one of the key nodes linking anxiety and depression [17]. Altogether, these findings suggest that across age and educational contexts, cooperative and supportive aspects of family functioning consistently occupy central positions in psychological networks. The central roles of FC2 and FC4 in this study are thus validated across different samples, reinforcing the idea that family functioning may represent a relatively consistent protective factor for university students’ emotional systems.

In the network analysis, BEI was applied to identify nodes that act as key links between different psychological domains. The results showed that BS4 (distractibility) had the highest BEI value, indicating its prominent bridging position across BFNE, self‐control, and family functioning. This pattern of associations may be interpreted from both short‐term and long‐term perspectives. According to the attentional control theory [54, 55], heightened BFNE consumes cognitive resources through excessive self‐focus and concerns about others’ evaluations. This may be related to reduced regulatory capacity, manifesting as distractibility and impaired cognitive control. From the perspective of social support theory [32, 33], family functioning plays a protective role in the development of executive function and attentional regulation. By contrast, low levels of family functioning reduce the ability to sustain attention and maintain self‐regulation. Consequently, BS4 appears to be associated with both attentional disruption and family‐related factors associated with BFNE and the long‐term regulatory influence of family functioning. This is consistent with its role as a bridging node between these domains. Empirical findings support this interpretation. A study of university students in Hong Kong found that self‐control buffered the emotional effects of negative evaluation. In contrast, distractibility, as a marker of low self‐control, weakened regulatory processes and intensified negative affect [56]. Another network analysis with adolescents showed that low self‐control behaviors acted as a central pathway in the psychological network, linking suicidal self‐injury and problematic smartphone use [57]. This finding suggests that deficits in self‐control are associated with failures in emotion regulation and may reflect a shared association pattern underlying multiple maladaptive behaviors. Additionally, research on the network structure of smartphone use and exercise procrastination identified self‐control as a critical bridging variable, particularly in coordinating processes between attentional regulation and impulsive tendencies [28]. Altogether, these findings further support the identification of BS4 (distractibility) as a bridge node in this study. This suggests that attentional control difficulties may be associated with multiple domains, including negative emotions, self‐regulation, and family functioning. The bridge centrality analysis also identified BS1 (self‐control) and BS7 (impulsivity) as the second‐ and third‐most influential bridge nodes, respectively. BS1 reflects individuals’ perceived control over their behaviors and emotions, acting as an essential buffer between evaluative pressure and family functioning. In contrast, BS7 captures impulsive tendencies such as impatience and lack of planning. This may amplify the interplay between negative emotions and low‐income family functioning [58, 59]. These findings align with recent network studies on university student populations, consistently emphasizing the cross‐domain importance of self‐control traits in anxiety, emotion regulation, and social functioning [23]. Collectively, the results highlight the bridging significance of self‐control–related characteristics, with attentional control emerging as particularly salient for understanding the interplay between emotional regulation processes and family influences.

In the flow network constructed in this study, adaptive emotion regulation strategies (ER1) and maladaptive emotion regulation strategies (ER2) were set as entry points to examine the pattern of conditional associations through which these strategies interact with other psychological variables. Notably, family functioning nodes showed more connections with adaptive strategies (ER1) than maladaptive strategies (ER2), suggesting that family functioning may act more as a “resource‐enhancing” factor rather than merely a “risk‐buffering” mechanism. This pattern implies that supportive family environments may actively facilitate the development of adaptive CERSs. The results revealed that both strategies showed the strongest associations with BFNE12 (fear of negative evaluation), a finding of substantial psychological significance. BFNE12 reflects a heightened sensitivity to verbal mistakes in evaluative social situations. It represents a core feature of expressive anxiety, where individuals are overly concerned that speech errors will lead to negative evaluation. Its strong associations with both ER1 and ER2 suggest that BFNE12 may act as a central node linking different strategies, suggesting that it is closely associated with multiple regulation strategies within the network. Specifically, whether individuals use adaptive strategies such as cognitive reappraisal or maladaptive strategies such as suppression and avoidance, these strategies show shared associations with concerns about “saying something wrong,” thereby positioning expressive anxiety as one of the most strongly connected components within the network within the regulatory system [60–62]. This conclusion is supported by research showing that “catastrophizing” and “other‐blame” act as key bridging nodes linking alexithymia and emotion regulation strategies in a network model [29]. Although their study addressed a different population, the results similarly highlight how specific cognitive processing patterns are closely associated with the selection of strategies. Building on these findings, the present study identifies fear of verbal mistakes (BFNE12) as a key node that is associated with multiple regulatory pathways, highlighting its strong connectivity with multiple regulation‐related components within the network. From the perspective of emotion regulation theory [30, 31], in high‐stakes social situations, concern about “saying something wrong” often triggers defensive responses, typically activating maladaptive strategies (e.g., suppression, denial, or avoidance) to reduce anxiety in the short term. However, individuals with greater regulatory flexibility or contextual awareness may subsequently shift toward more adaptive strategies such as cognitive reappraisal, reframing the event in a less‐threatening manner. Thus, BFNE12 can be seen as an early‐related cognitive component within the network in the regulation process, one that is associated with both maladaptive and adaptive regulation strategies within the network. This interpretation is broadly consistent with prior findings. Studies of Chinese college students have shown that cognitive reappraisal not only alleviates anxiety but also plays a coordinating role in broader emotion networks [63]. Similarly, recent research suggests that in evaluative social contexts, maladaptive strategies such as suppression tend to be activated first. In contrast, the effective use of reappraisal requires stronger contextual appraisal and higher self‐efficacy [64]. Our findings are consistent with these perspectives, suggesting that BFNE12 is consistently connected with multiple regulation strategies within the network, underscoring its central role in various strategies. Moreover, network analyses conducted during public health emergencies have shown that maladaptive strategies such as catastrophizing and self‐blame occupy central positions in psychological symptom networks and are strongly associated with anxiety and depression. Unlike those studies, which emphasized strategy‐centered outcomes, the present study highlights BFNE12 as a node that shows strong associations with multiple regulation strategies within the network. These findings may provide tentative implications for future research directions: Addressing expressive anxiety, particularly concerns about verbal mistakes, may represent a potential point of interest for future investigation for promoting healthier regulatory pathways. Specifically, future studies may further explore whether approaches such as cognitive reappraisal training or exposure‐based methods are associated with changes in these patterns.

## 5. Conclusions

Based on a large sample of Chinese university students, this study constructed a psychological network involving four core constructs: fear of negative evaluation, family functioning (FC), self‐control (BS), and CERSs. The findings identified BS4 (distractibility) and BS1 (self‐control) as prominent bridging nodes, linking otherwise distinct psychological domains. This suggests that attentional control and executive functioning may be important factors associated with multiple psychological domains and may be relevant for future research examining these associations. In addition, the flow network analysis revealed that BFNE12 (fear of saying something wrong) showed the strongest associations with both adaptive and maladaptive emotion regulation strategies, suggesting its potential importance in expressive social anxiety. Future research may further examine how expressive anxiety is associated with different coping and regulation patterns, which may be relevant to reducing fear of negative evaluation and to adaptive coping in evaluative contexts. Taken together, this study provides new insights into the association patterns connecting fear of negative evaluation, self‐regulation, and family functioning. Future research should adopt longitudinal designs and diverse cultural or educational samples to further examine these association patterns and explore potential developmental processes through which key network nodes shape the psychological adjustment of university students.

## 6. Limitations

This study has several limitations that should be acknowledged.

First, the cross‐sectional design precludes any inference of temporal or causal relationships among variables. The estimated network reflects conditional associations at a single time point, and the observed connections should not be interpreted as directional or predictive effects. Longitudinal or experimental studies are needed to further examine the stability and potential dynamic processes underlying the identified network structure.

Second, the sample was obtained through convenience sampling from three universities in Wuhan, which may limit the representativeness and generalizability of the findings to broader student populations or other cultural contexts. In addition, the sample was predominantly female. Although this gender distribution reflects the characteristics of medical universities in China, potential gender differences in psychological constructs such as fear of negative evaluation, family functioning, and self‐control cannot be ruled out. In the present study, gender was not included as a covariate, and network invariance across subgroups was not examined. Moreover, the measurement invariance of the employed scales across demographic groups was not formally tested. Given that the present study involved item‐level interpretations, future research should further evaluate whether the measurement properties and network structures remain stable across different subpopulations.

Third, although several demographic and contextual variables (e.g., gender, socioeconomic background, and academic characteristics) were collected, they were not incorporated into the network estimation or examined in additional analyses. As a result, the observed network structure may be influenced by unmeasured or uncontrolled confounding factors. Future studies are encouraged to assess the robustness of network structures by incorporating covariates or conducting subgroup analyses.

Fourth, although most variables were measured using Likert‐type or ordinal response formats, the primary analyses were conducted within a GGM framework with nonparanormal transformation. To address this issue, we conducted additional sensitivity analyses using ordinal‐based correlation estimation (cor_auto) under the same EBICglasso framework. The results demonstrated a high degree of consistency between the ordinal model and the primary model in terms of the overall edge structure and centrality indices, supporting the robustness of the main findings. However, some variability was observed in the relative ranking of less central nodes, suggesting that these estimates may be somewhat sensitive to the choice of the estimation method. Future studies may further extend these analyses using alternative approaches specifically designed for ordinal data.

Fifth, the use of the Family APGAR, while efficient and widely adopted, involves only five items and may not fully capture the structural and functional complexity of family systems, such as communication patterns, role distribution, or authority dynamics. Future research may benefit from incorporating more comprehensive and multidimensional assessments of family functioning.

Finally, although centrality and bridge metrics provide useful insights into the relative importance of nodes within the network, these indices should not be directly interpreted as definitive intervention targets, particularly in cross‐sectional designs. The practical implications discussed in this study should therefore be considered preliminary and hypothesis‐generating. Future longitudinal and intervention studies are needed to further examine whether targeting specific nodes is associated with meaningful improvements in psychological outcomes.

## Funding

No funding was received for this manuscript.

## Conflicts of Interest

The authors declare no conflicts of interest.

## Supporting Information

Additional supporting information can be found online in the Supporting Information section.

## Supporting information


**Supporting Information 1** Material S1: STROBE checklist for reporting observational studies.

**Supporting Information 2** Material S2: This Supporting Information presents all scale items included in the study and their corresponding node abbreviations used in the network analyses.

**Supporting Information 3** Material S3: This Supporting Information presents the bootstrap analyses used to evaluate the reliability and stability of the network model, including edge‐weight confidence intervals, edge‐weight difference tests, and node expected influence difference tests.

## Data Availability

The data that support the findings of this study are available in the Supporting Information of this article.
