# Supplementary material for: Bridging Fear of Negative Evaluation and Cognitive Emotion Regulation Strategies: A Network Perspective on the Roles of Family Functioning and Self‐Control
Source: Depress Anxiety. 2026 May 29;2026:1391628. doi: 10.1155/da/1391628 (PMC13239512; doi:10.1155/da/1391628)

Figure 1.Bootstrap confidence intervals for the edge weights of the network model.

This section presents the 95% bootstrap confidence intervals for the edge weights of the network model. These confidence intervals help assess the stability and reliability of the network connections.

Figure 1: Bootstrap Confidence Intervals for Edge Weights


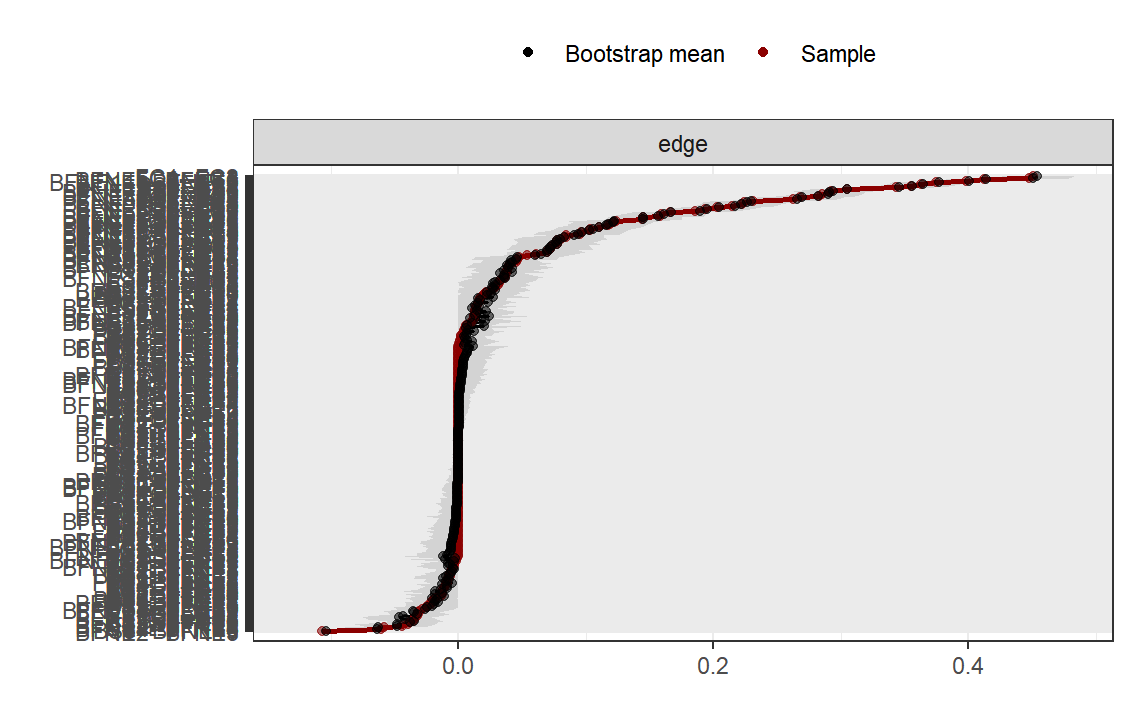


Figure 2.Edge weights identified as significantly different via bootstrap difference test.

This section shows the edge weights that were identified as significantly different through the bootstrap difference test. This analysis is crucial for identifying important structural relationships in the network.

Figure 2: Edge Weights Identified as Significantly Different via Bootstrap Difference Test


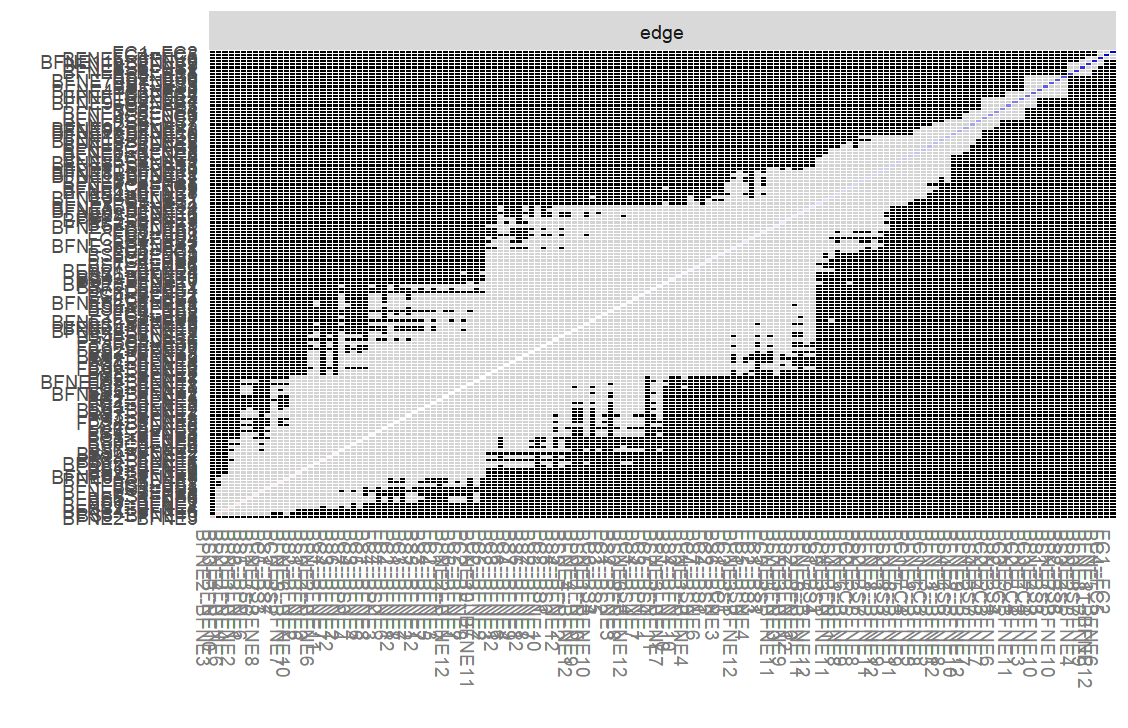


Figure 3.Bootstrap difference test for node expected influence centrality indices.

This section displays the results of the bootstrap difference test for node expected influence centrality indices. These tests help identify the most influential nodes in the network.

Figure 3: Bootstrap Difference Test for Node Expected Influence Centrality Indices


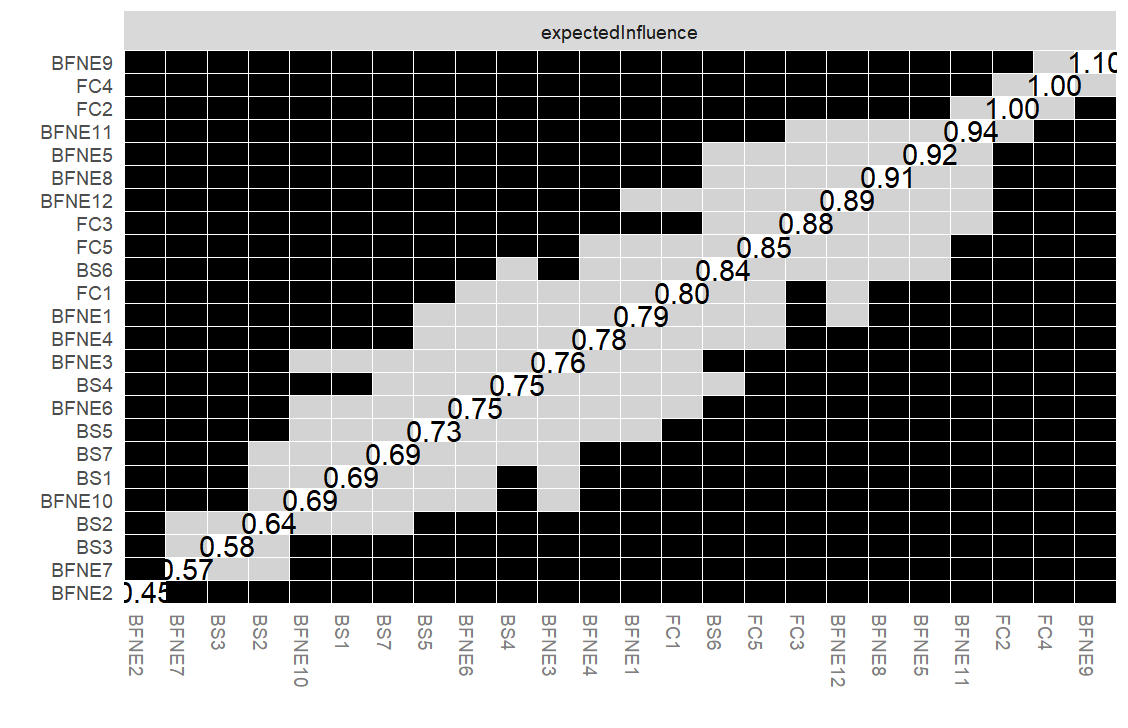

Supplement: Supplementary file 1 — Supporting Information 1 Material S1: STROBE checklist for reporting observational studies. [file DA-2026-1391628-s002.docx]
