# Supplementary material for: Bridging Fear of Negative Evaluation and Cognitive Emotion Regulation Strategies: A Network Perspective on the Roles of Family Functioning and Self‐Control
Source: Depress Anxiety. 2026 May 29;2026:1391628. doi: 10.1155/da/1391628 (PMC13239512; doi:10.1155/da/1391628)
Supplement: Supplementary file 2 — Supporting Information 2 Material S2: This Supporting Information presents all scale items included in the study and their corresponding node abbreviations used in the network analyses. [file DA-2026-1391628-s001.pdf]

Brief Fear of Negative Evaluation Scale (BFNE)		
**Item abbreviation**	**Item content**	**Items context**
**BFNE1**	Even though I know it does not really matter, I worry about what others think of me.	Worry about judgment
**BFNE2**	Even when I know others are forming an unfavorable impression of me, it does not bother me.	Indifference to negative views
**BFNE3**	I am often afraid that others will notice my shortcomings.	Fear of criticism
**BFNE4**	I am not concerned about the impression I make on others.	Unconcerned with impressions
**BFNE5**	I am afraid that others will disapprove of me.	Fear of disapproval
**BFNE6**	I am afraid that others will find fault with me.	Fear of mistakes
**BFNE7**	I am not bothered by what others think of me.	Unbothered by opinions
**BFNE8**	When talking with others, I worry about what they may think of me.	Worry in conversations
**BFNE9**	I am usually concerned about the impression I make on others.	Constant concern about impressions
**BFNE10**	When I know someone is evaluating me, it does not bother me.	Unfazed by evaluation
**BFNE11**	Sometimes I think I care too much about what others think of me.	Overly concerned with opinions
**BFNE12**	I often worry that I will say or do the wrong thing.	Fear of saying something wrong

The Brief Self-Control Scale (BSCS)		
**Item abbreviation**	**Item content**	**Items context**
**BS1**	I am good at resisting temptation.	Self-control
**BS2**	I sometimes do things that bring me pleasure but are harmful to me.	Impulsivity
**BS3**	People say that I have strong self-discipline.	Discipline
**BS4**	Sometimes I get distracted by enjoyable activities and fail to complete tasks on time.	Distractibility
**BS5**	I am able to work efficiently toward long-term goals.	Goal-orientation
**BS6**	Sometimes I cannot resist doing things that I know are wrong.	Temptation-prone
**BS7**	I often act without thinking things through.	Impulsiveness

The Family APGAR Questionnaire (APGAR)		
**Item abbreviation**	**Item content**	**Items context**
**FC1**	I am satisfied with the help I receive from my family when I have problems.	Adaptation
**FC2**	I am satisfied with the way my family discusses things with me and shares problems with me.	Partnership
**FC3**	I am satisfied that my family accepts and supports my wishes to engage in new activities or pursue personal growth.	Growth
**FC4**	I am satisfied with the way my family expresses love and responds to my emotions (such as happiness, anger, sadness, and joy).	Affection
**FC5**	I am satisfied with the way my family and I spend time together.	Resolve

The Chinese Version of the Cognitive Emotion Regulation Questionnaire (CERQ-C)			
**Item abbreviation**		**Item content**	**Items context**
**ER1**	**CERQ1**	I feel that I am responsible for what has happened.	Self-blame1
	**CERQ2**	I think that the main cause of the situation lies within myself.	Self-blame2
	**CERQ3**	I think that I have to accept what has happened.	Acceptance1
	**CERQ4**	I feel that I have to accept the situation.	Acceptance2
	**CERQ5**	I often think about how I feel about what I have experienced.	Rumination1
	**CERQ6**	My thoughts are constantly occupied by what I have experienced and how I feel about it.	Rumination2
	**CERQ7**	I think about pleasant things that are unrelated to what has happened.	Positive refocusing1
	**CERQ8**	I think about positive things instead of what has just happened.	Positive refocusing2
	**CERQ9**	I think about how to change the situation.	Refocus on planning1
**ER2**	**CERQ10**	I think about the best way to deal with the situation.	Refocus on planning2
	**CERQ11**	I think that I can learn something from the situation.	Positive reappraisal1
	**CERQ12**	I think that the situation has made me a stronger person.	Positive reappraisal2
	**CERQ13**	I think that things could have been much worse.	Putting into perspective1
	**CERQ14**	I tell myself that there are worse things in life.	Putting into perspective2
	**CERQ15**	I keep thinking about how terrible what I have experienced is.	Catastrophizing1
	**CERQ16**	I keep thinking about how awful the situation was.	Catastrophizing2
	**CERQ17**	I feel that others are responsible for what has happened.	Other-blame1
	**CERQ18**	I think that the main cause of the situation lies with others.	Other-blame2
